# The role of kynurenines in migraine-related neuroimmune pathways

**DOI:** 10.1186/s10194-024-01833-z

**Published:** 2024-08-07

**Authors:** Tamás Körtési, Gábor Nagy-Grócz, László Vécsei

**Affiliations:** 1 Department of Theoretical Health Sciences and Health Management, Faculty of Health Sciences and Social Studies, University of Szeged, Temesvári krt. 31, Szeged, H-6726 Hungary; 2 HUN-REN-SZTE Neuroscience Research Group, Hungarian Research Network, Danube Neuroscience Research Laboratory, University of Szeged (HUN-REN-SZTE), Tisza Lajos krt. 113, Szeged, H- 6725 Hungary; 3 Preventive Health Sciences Research Group, Incubation Competence Centre of the Centre of Excellence for Interdisciplinary Research, Development and Innovation of the University of Szeged, Szeged, H-6720 Hungary; 4 Department of Neurology, Albert Szent-Györgyi Medical School, University of Szeged, Semmelweis u. 6, Szeged, H-6725 Hungary

**Keywords:** Migraine, Inflammation, Neuropeptides, Kynurenines, Neuroimmune process

## Abstract

Migraine, a primary headache disorder whose mechanism remains incompletely understood, appears to involve the activation of the trigeminovascular system (TS) during attacks. Research suggests that inflammatory processes mediated by the immune system may play a role in migraine pathophysiology. Neuroinflammation is often associated with migraine attacks, with cytokines serving as crucial mediators in the process. Elevated levels of pro-inflammatory cytokines, such as interleukin-1 beta (IL-1β), interleukin-6 (IL-6), and tumor necrosis factor-alpha (TNF-α), have been observed in the blood and cerebrospinal fluid of individuals experiencing migraine attacks. These cytokines have the capacity to sensitize pain pathways in the brain, thereby increasing sensitivity to pain stimuli. This phenomenon, known as central sensitization, is believed to contribute to the intensity and persistence of migraine pain. Kynurenines, endogenous mediators of glutamatergic mechanisms, can significantly influence the pathophysiology of primary headache disorders. The kynurenine system is collectively known as the kynurenine pathway (KP), which can act on multiple receptors, such as glutamate receptors, aryl hydrocarbon receptors (AhRs), G protein-coupled receptors 35 (GPR35), and α-7 nicotinic acetylcholine (α7 nACh) receptors. These receptors are also found on various cells of the immune system, so the role of the KP in the pathomechanism of primary headaches may also be mediated through them. In this review, our goal is to show a possible link between the receptors of the KP and immune system in the context of inflammation and migraine. Migraine research in recent years has focused on neuropeptides, such as calcitonin gene-related peptide (CGRP) and pituitary adenylate cyclase-activating polypeptide (PACAP) as potential pathogenic factors and possible therapeutic approaches. These peptides share many similarities in their characteristics and roles. For instance, they exhibit potent vasodilation, occur in both the peripheral and central nervous systems, and play a role in transmitting nociception and neurogenic inflammation. The investigation of potential connections between the aforementioned neuropeptides and the kynurenine pathway could play a significant role in uncovering the pathomechanism of migraine and identifying new drug candidates.

## Background

Migraine is a primary headache disorder marked by moderate to severe unilateral pain that pulsates or throbs, often accompanied by symptoms like nausea/vomiting or sensitivity to light and sound. Headaches can be triggered by various factors including weather, alcohol, stress, or hormonal fluctuations. The disease ranks among the most prevalent neurological conditions, carrying significant morbidity and correlating with a substantial economic burden [1, 2].

Migraine consists of four phases. The prodrome phase initiates up to 24 h before the headache occurs. An aura serves as an indication of an impending migraine headache, presenting with sensory, motor, and/or speech symptoms. This phase can endure for up to 60 min or as briefly as five. It is possible to experience both the aura and the headache simultaneously. A migraine headache typically persists for a duration ranging from 4 h to 72 h. The postdrome stage typically extends for a few hours to as long as 48 h. Symptoms resemble those of an alcohol-induced hangover, hence the term “migraine hangover” for this phase [3].

While there is ongoing debate about the precise pathophysiological mechanism of migraine, the prevailing theory suggests it is a disorder impacting sensory processing in the brain.

Towards the end of the 20th century, Moskowitz and his colleagues proposed the trigeminovascular hypothesis of migraine, emphasizing the crucial involvement of the trigeminal nerve and its axonal projections containing vasoactive neuropeptides to the meninges and blood vessels [4]. The correlation between the activation of both the peripheral and central branches of the trigeminovascular system (TS) with cortical spreading depression and the activity of specific brainstem nuclei leads us to the conclusion that migraine could be attributed to a disrupted function of the neuronal components within the TS, the brainstem, and the cortex. At the core of this mechanism lies the activation and senzitization of the TS [2]. Nowadays it is believed that headache primarily arises from TS activation, leading to neurogenic vasodilation and meningeal inflammation [5]. It is clear that inflammation contributes to headache onset. The fact that non steroidal anti-inflammatory drugs provide only partial relief from headaches suggests that prostaglandins are involved in sensitizing nociceptors. The activation of mast cells already present in the meninges, along with the subsequent release of inflammatory molecules like interleukin-1β (IL-1 β), interleukin-6 (IL-6), tumor necrosis factor-α (TNF- α), and various chemokines, is suggested to significantly influence the development of migraine headaches. Additionally, the release of cytokines by glial cells is also believed to contribute to the mechanism of migraine. Studies indicate that cortical spreading depression can trigger inflammasome activation within the brain tissue, further reinforcing this involvement [6–8]. The vascular theory will likely remain prominent for a few more years, further refining peripheral drug targets with increasing precision. Although the source of the pain, the ultimate trigeminal nociceptor, is located around the meningeal (and probably also cortical) vessels, the pathogenesis of migraine is not primarily due to a defective (cranial) vascular system. For this nociceptor to be activated, a central generator, specifically the limbic system including the hypothalamus, is required [9]. The latest MRI studies suggest that the limbic system and sensory system play an important role in the pathophysiology of migraines. In this study, it was demonstrated that the limbic regions, especially the orbitofrontal cortex and temporal pole, showed an expansion of the connectome manifold. These findings may suggest greater differentiation between connectivity within the limbic regions compared to the rest of the brain. Conversely, the sensorimotor regions exhibited contractions in manifold eccentricity, indicating more integrated connectivity patterns within the brain [10].

**Fig. 1:**
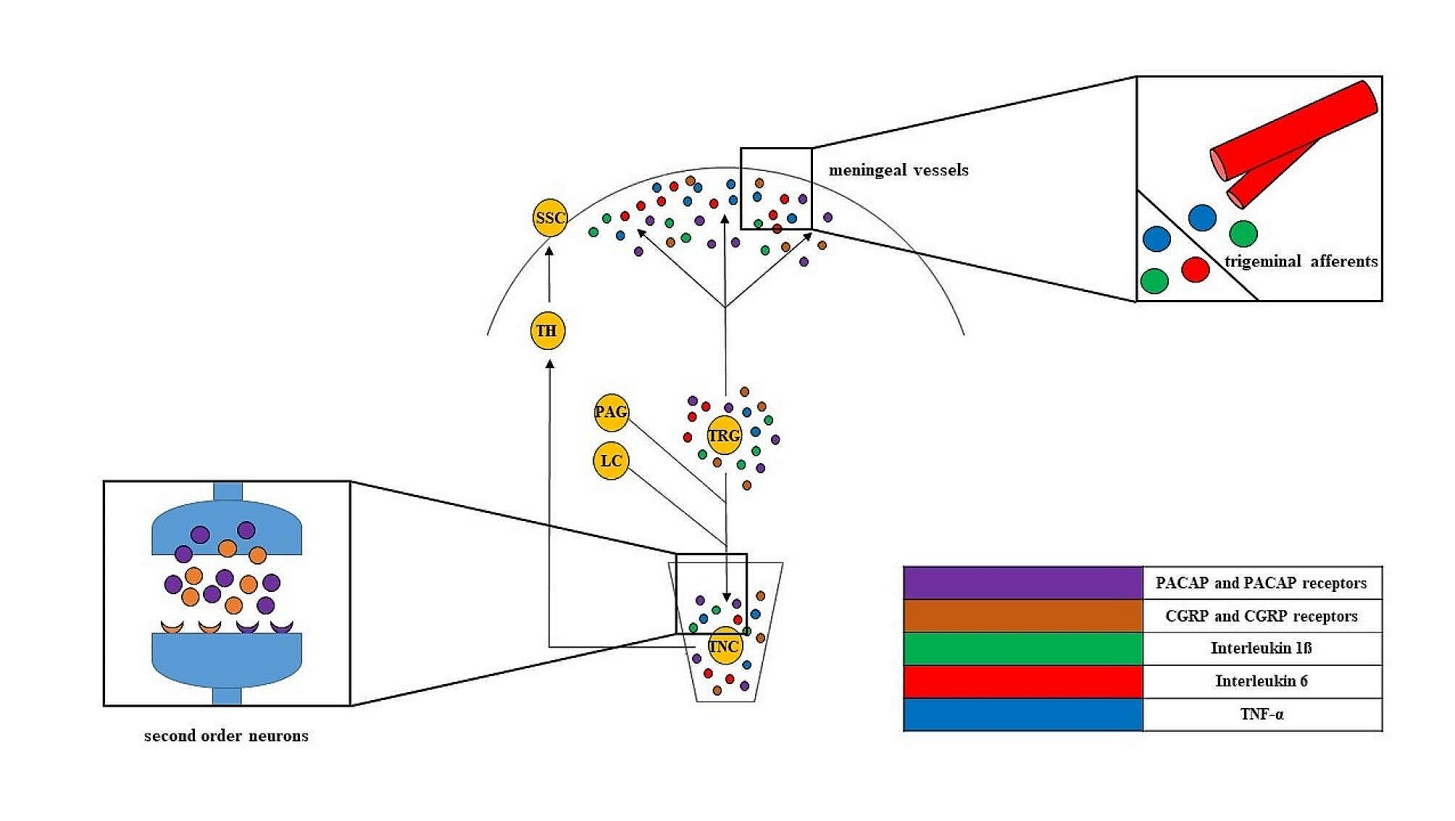
Theoretical involvement of inflammatory molecules in migraine attack: When immune cells become activated, they modify the microenvironment around the TS by releasing inflammatory substances cytokines, chemokines and neuropeptides. These substances prompt the widening of blood vessels in the dura mater and affect the integrity of tight junctions between endothelial cells. Through this process, activated trigeminal neurons send signals to higher brain regions, leading to the sensation of pain and heightened sensitivity. TNC: nucleus trigeminus caudalis, TRG: trigeminal ganglion PAG: periaqueductal grey, LC: locus coeruleus TH: thalamus, SSC: somatosensory cortex

Recent evidence indicates that significant contributors to headache development are the neuropeptides calcitonin gene-related peptide (CGRP) and pituitary adenylate cyclase activating polypeptide (PACAP) [11, 12]. These peptides share many similarities in their characteristics and roles. For example, they act as potent vasodilators, are found in both the peripheral and the central nervous systems (CNS), and contribute to the transmission of pain sensation and neurogenic inflammation [13]. Consequently, they have become significant focal points in the therapeutic advancements for migraine. In 2004, the first CGRP receptor antagonists demonstrated effective migraine termination in humans. However, currently, numerous other anti-CGRP treatments are either in clinical trials or undergoing development. PACAP1–38 also plays a pivotal role in migraine, as suggested by various preclinical and clinical investigations [14–16] However, there are fewer confirmed outcomes specifically focused on PACAP1–38 antibody therapies. PACAP receptor PAC1 monoclonal antibody (mAb), AMG 301, showed no migraine prevention benefit, while the PACAP ligand mAb, Lu AG09222, notably reduced monthly migraine days in a phase 2 trial. The similar behavior of these peptides raises the possibility that anti-PACAP1–38 treatments could offer a therapeutic benefit for migraine sufferers who do not respond to anti-CGRP therapies [17–19] [insert Fig. 1.].

Despite intensive research, the exact sequence of events during headache episodes and the relative significance of central and peripheral mechanisms remain unclear. With the exception of recently approved mAb therapies, much of the preventive treatment relies on empirical observations rather than a comprehensive understanding of the pathophysiology.

While migraine is typically characterized by a homogeneous clinical symptom complex, it is most often underpinned by heterogeneous molecular mechanisms. In a previous study, we detected 144 genes with differing expression levels in peripheral blood mononuclear cells between samples from individuals with headache and those without, and 163 genes between symptom-free patients and controls. Network analysis indicated enrichment in pathways related to inflammation, cytokine activity, and mitochondrial dysfunction in both headache-afflicted and headache-free samples compared to controls [20].

The immune system’s involvement in migraine has garnered significant attention in recent research. Emerging evidence suggests that immune dysregulation and inflammation may play key roles in migraine pathophysiology. Specifically, immune cells, such as mast cells and microglia, have been implicated in triggering and perpetuating migraine attacks. Mast cells release inflammatory mediators such as histamine and cytokines in response to various triggers. These mediators can activate and sensitize nearby pain-sensing nerve fibers, contributing to the initiation and propagation of migraine pain. Microglia become activated in response to inflammatory signals. Once activated, microglia release pro-inflammatory molecules and neuroexcitatory substances, further promoting neuronal sensitization and pain amplification in migraine. Additionally, cytokines and other immune modulator molecules have been found to be dysregulated in migraine patients, indicating a broader immune system involvement in the disorder. Understanding the intricate interplay between the immune system and migraine is crucial for developing targeted therapies that can effectively modulate immune responses and alleviate migraine symptoms [21–23].

## The structure of the kynurenine pathway (KP)

**Fig. 2:**
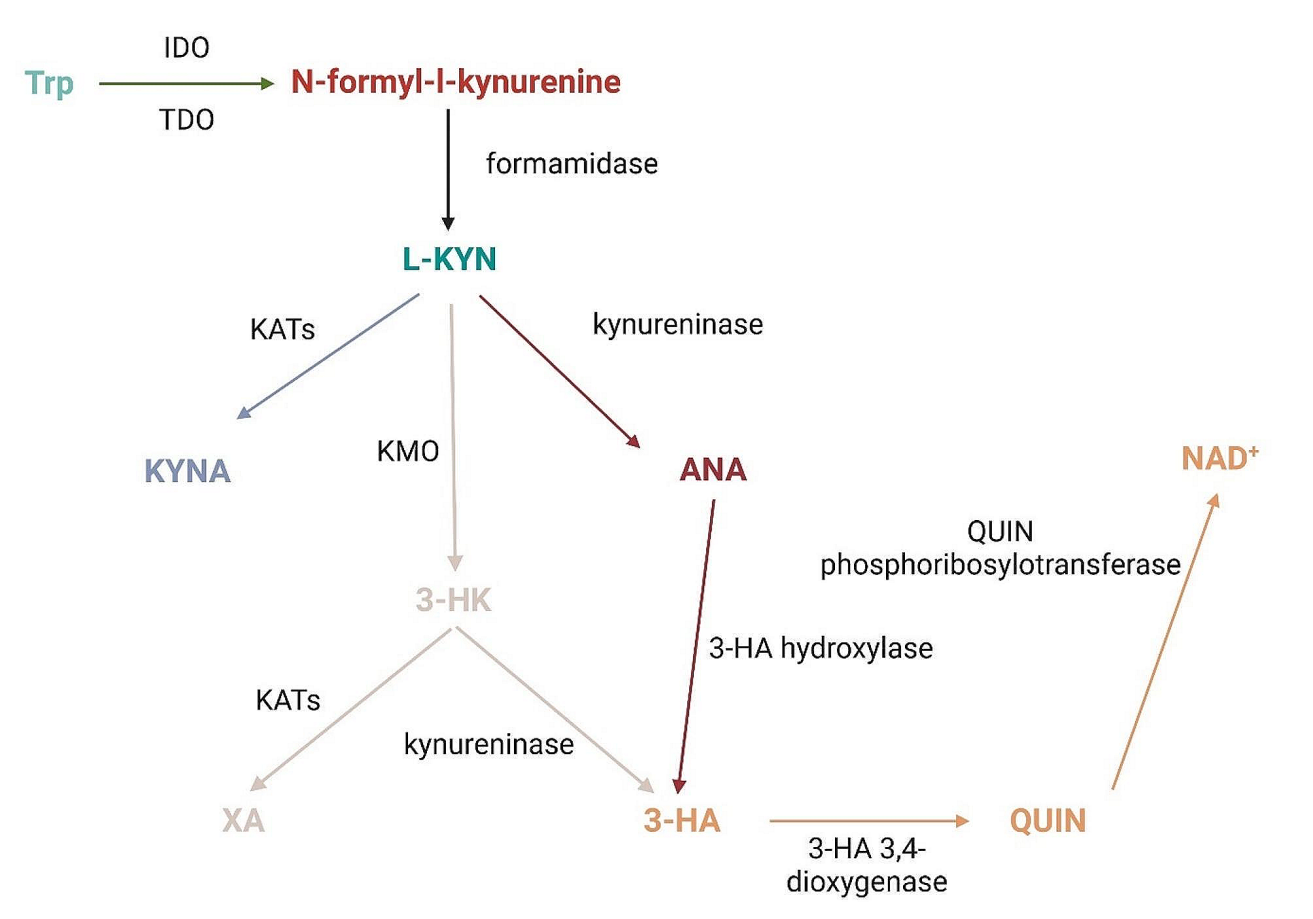
The kynurenine pathway. This figure shows the main metabolites and enzymes of the KP. Abbreviations: 3-HA—3-hydroxyanthranilic acid, 3-HK—3-hydroxykynurenine, 5-HT—serotonin, ANA—anthranilic acid, KYNA—kynurenic acid, L-KYN—L-kynurenine, NAD+—nicotinamide adenine dinucleotide, QUIN—quinolinic acid, Trp—tryptophan, XA—xanthurenic acid, IDO—indoleamine 2,3-dioxygenase, TDO—tryptophan 2,3-dioxygenase, KATs—kynurenine aminotransferases, KMO—kynurenine 3-monooxygenase

Tryptophan (Trp) is a crucial amino acid, essential for brain function, serving as the precursor for serotonin (5-HT). However, over 90% of Trp in mammalian cells metabolizes in the KP rather than towards 5-HT (Fig. 1). Kynurenic acid (KYNA), a prominent KP product discovered by Justus von Liebig in 1853, acts as an endogenous glutamate receptor antagonist with neuroprotective effects. KYNA is synthesized by kynurenine aminotransferases (KATs) from L-kynurenine (L-KYN), derived from N-formylkynurenine through the action of formamidase enzyme. Notably, L-KYN can convert to anthranilic acid (ANA) via kynureninase (3-HAO) and to 3-hydroxykynurenine (3-HK) through kynurenine 3-monooxygenase (KMO), in addition to KYNA. KMO, a mitochondrial protein in eukaryotic cells, resides in the outer mitochondrial membrane. ANA can further transform into 3-hydroxyanthranilic acid (3-HAA) with the assistance of 3-hydroxyanthranilic acid hydroxylase. Additionally, 3-HK may convert to 3-HAA via kynureninase, or to xanthurenic acid. 3-HAA, when exposed to 3-hydroxyanthranillic acid 3,4-dioxygenase, can further convert into quinolinic acid (QUIN). The final step in the KP involves the transformation of QUIN into nicotinamide adenine dinucleotide (NAD^+^) through quinolinic acid phosphoribosyl transferase. NAD^+^ plays a crucial role in mitochondrial energy management and redox reactions. In contrast to KYNA, QUIN serves as an endogenous glutamate receptor agonist produced by microglia. QUIN may induce inflammation, and neuronal dysfunction in the CNS [24]. Research indicates that KP enzymes and substances play pivotal roles in the pathomechanisms of neurological disorders, such as depressive disorders [24, 25], schizophrenia [26], and migraine [27, 28] [Insert Fig. 2.].

### Receptors of the KP and their roles in the immune system

**Fig. 3:**
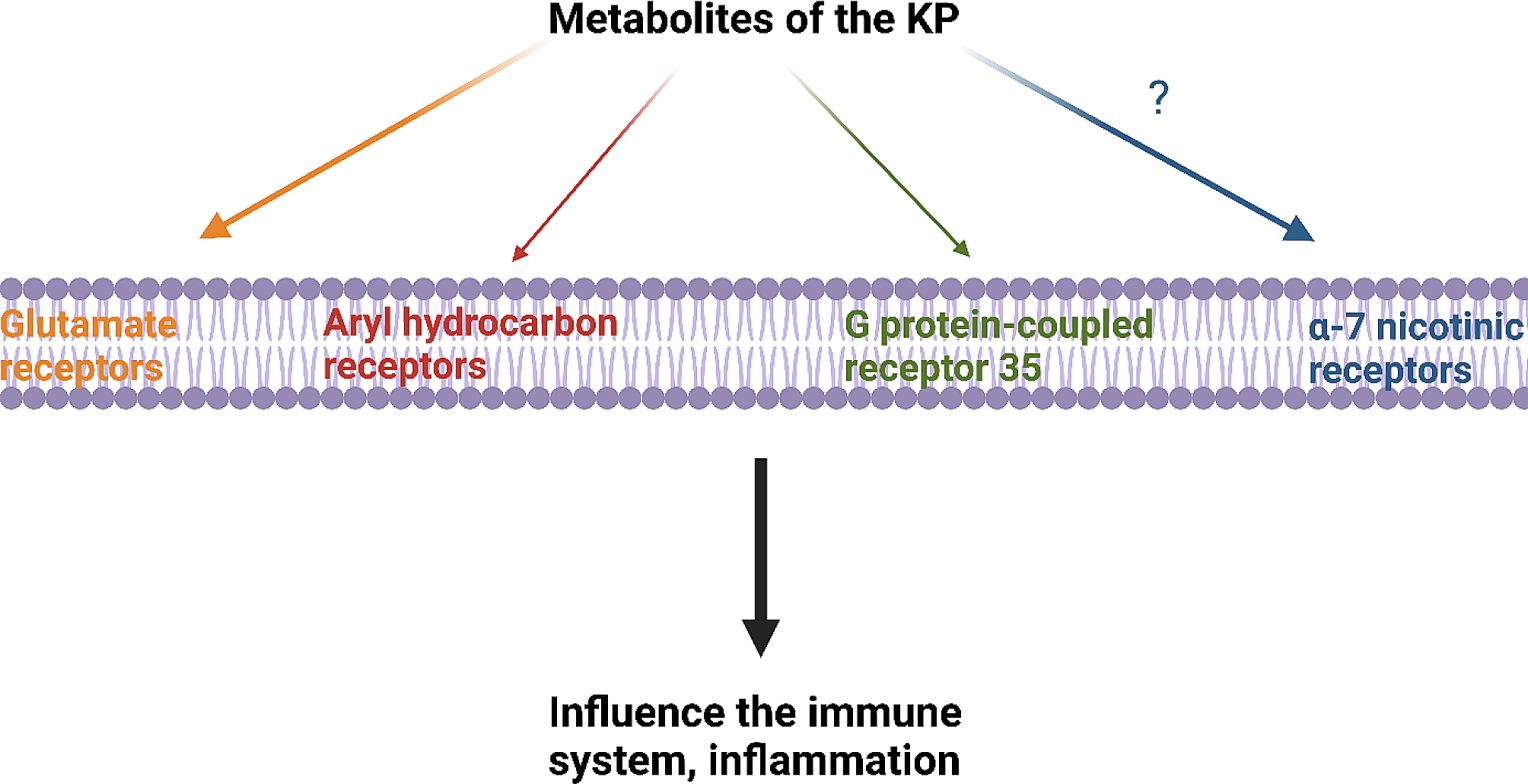
Possible receptors where KP metabolites could bind. The figure offers a summary of potential receptors in the immune system that could interact with the KP. Recent research has brought up questions regarding its impact on the alpha-7 nicotinic receptors

Kynurenines exert their effects on various receptors, including dose-dependent interactions with glutamate receptors, aryl hydrocarbon receptors (AhRs), G protein-coupled receptors 35 (GPR35), and α-7 nicotinic acetylcholine receptors (α7 nAChRs) (Fig. 3) [Insert Fig. 3.].

### Glutamate receptors

Glutamate receptors constitute a class of neurotransmitter receptors present in the CNS of animals, including humans. Being the most abundant excitatory neurotransmitter in the brain, glutamate and its receptors play a fundamental role in diverse aspects of neuronal communication, and synaptic plasticity. Two major categories encompass glutamate receptors: ionotropic glutamate receptors, functioning as ligand-gated ion channels, mediating fast synaptic transmission, and including N-methyl D-aspartate (NMDA receptors), α-amino-3-hydroxy-5-methyl-4-isoxazole propionic acid AMPA receptors, and kainate receptors. The other types of glutamate receptors are metabotropic glutamate receptors, which are G protein-coupled receptors linked to ion channels through intracellular signaling pathways, modulating synaptic transmission, and influencing neuronal excitability. KYNA acts as an antagonist at the strychnine-insensitive glycine-binding site of NMDA receptors at low doses [29] and inhibits the glutamate-binding site at higher doses [30]. Additionally, KYNA has antagonistic effects on kainate and AMPA receptors, with its impact on AMPA receptors being concentration-dependent, stimulating them at nanomolar and micromolar concentrations while inhibiting them between micromolar and millimolar concentrations [31, 32]. KYNA is often referred to as a “Janus-faced” molecule because, like the Roman god Janus, it has two distinct and often contradictory aspects or functions [33]. Taken together, KYNA exhibits dual properties within biological systems.

Glutamate receptors are also found on various peripheral immune cells, including T cells [34], macrophages [35, 36], and dendritic cells [37]. Inflammation can lead to dysregulation of glutamate signaling, which may contribute to neuronal damage and neuroinflammation. Glutamate receptors, particularly NMDA receptors, are expressed on microglial cells [38], the resident immune cells of the CNS. Furthermore, activation of these receptors can modulate microglial function [39], influencing the release of pro-inflammatory cytokines and reactive oxygen species (ROS) [40]. On the other hand, glutamate receptors on astrocytes, another type of glial cell, can also modulate inflammatory responses. In addition to this, glutamate signaling in astrocytes can lead to the release of inflammatory mediators such as cytokines and chemokines, contributing to neuroinflammation [41]. To bring together, glutamate receptors are found within the immune system, and the KP plays a role in regulating the immune system via these receptors.

### Aryl hydrocarbon receptors

AhRs, a family of proteins found in various species, including humans, are ligand-activated transcription factors that can bind to aromatic hydrocarbons like dioxins and polycyclic aromatic hydrocarbons. AhRs also regulate the expression of genes related to indoleamine 2,3-dioxygenase (IDO), and they have a crucial role in inflammation processes [42]. The activation of AhRs leading to the induction of IDO1 holds significance, as IDO1 plays a crucial role in producing L-KYN through the breakdown of Trp, thereby establishing a positive feedback loop. Furthermore, AhRs have been observed to positively control the expression of SLC7A5 [43, 44], which is a transporter of essential amino acids. The upregulation of SLC7A5 in response to AhR activation by L-KYN is likely to enhance the entry of L-KYN into cells, presenting an additional feed forward mechanism for AhR pathway activation in the context of inflammation. Furthermore, kynurenine’s activation of AhR possesses immunosuppressive qualities affecting both innate and adaptive immunity. When kynurenine binds to AhRs, it suppresses inflammatory reactions triggered by LPS in macrophages and enhances endotoxin tolerance, as evidenced by studies [45, 46]. Additionally, in the presence of transforming growth factor-β, L-KYN diminishes the differentiation of T cells into highly inflammatory Th17 cells while promoting the formation of Foxp3 + regulatory T cells, as demonstrated by Mezrich’s team [47]. In addition to this, the AhR pathway likely plays a role in various immune processes crucial for the host’s intestinal homeostasis and the maintenance of an optimal microbiome [48]. Additionally, it may contribute to innate immune reactions against microbial infiltration of barrier tissues [49]. Moreover, AhRs might influence the differentiation and functionality of both CD4 + and CD8 + T cells [50], potentially influencing chronic autoimmune damage to CNS neurons [51]. As demonstrated above, the function of AhRs in the immune system is already comprehensively understood, and the KP might have the capacity to impact immunological processes via their modulation.

### GPR35

Members of the G protein-coupled receptor family, GPR35 exhibit significant expression within various immune cells including eosinophils, monocytes, and natural killer-like T cells, hinting at their potential physiological significance in these cell types [52]. Moreover, their upregulation in activated neutrophils may potentially enhance their migratory capabilities [53]. Studies have shown that GPR35 activation can influence the production of pro-inflammatory cytokines, such as TNF-α, IL-6, and IL-1β [54]. Activation of GPR35 has been linked to both pro-inflammatory and anti-inflammatory effects, depending on the cellular context and the specific ligands involved. It is well-known that 5-HT and melatonin, which are metabolites of Trp, are widely recognized as ligands for GPRs. In the case of GPR35, kynurenines may bind to these receptors and initiate signaling cascades within cells, and activate the receptors in micromolar concentrations [55]. Despite the progress made in understanding the interaction between kynurenines and GPR35, there are still many unanswered questions. Further research is needed to elucidate the specific mechanisms underlying their effects and to determine their potential therapeutic applications. Additionally, the development of selective ligands targeting GPR35 could help dissect their physiological roles and facilitate the translation of findings into clinical interventions.

### α7 nAChRs

α7 nAChRs, a type of nicotinic acetylcholine receptors, play a crucial role in neurotransmission within the CNS. Nicotinic receptors respond to the neurotransmitter acetylcholine with their name derived from the term nicotine. The effects of KYNA on α7 nAChRs have been put into question, as Stone’s research suggests conflicting results on this interaction [56]. Activation of the α7 nAChRs has been associated with potent anti-inflammatory effects, since these receptors are located in macrophages [57], dendritic cells [58], T-cells [59], B-cells [60], natural killer cells [61], microglia [62], and astrocytes [63]. Stimulation of these receptors by their endogenous ligand, acetylcholine, or by other agonists can lead to the suppression of pro-inflammatory cytokine production, including TNF-α [64], IL-1β [65], IL-6 [66], and interferon-gamma (IFN-γ) [67]. This anti-inflammatory response helps to dampen excessive immune activation and tissue damage during inflammation. One of the key mechanisms by which α7 nAChRs exert their anti-inflammatory effects is through the inhibition of nuclear factor-kappa B (NF-κB) signaling. NF-κB is a transcription factor that regulates the expression of many pro-inflammatory genes. Activation of α7 nAChRs inhibits NF-κB activation [68], thereby reducing the expression of pro-inflammatory cytokines and chemokines. Furthermore, α7 nAChRs activation influences the function of various immune cells involved in inflammation. It can modulate the activity of macrophages and monocytes, leading to a shift from a pro-inflammatory M1 phenotype to an anti-inflammatory M2 phenotype [69]. Additionally, α7 nAChRs activation can suppress the activation and proliferation of T cells [70] and inhibit the maturation and activation of dendritic cells [71, 72], thus regulating adaptive immune responses. In addition to their expression on immune cells, α7 nAChRs are also present on neurons in the peripheral nervous system, and the CNS [73]. Activation of α7 nAChRs on neurons can modulate neurotransmitter release, including the release of acetylcholine itself [74, 75], leading to the activation of the cholinergic anti-inflammatory pathway. This pathway involves the release of acetylcholine, which then acts on α7 nACh receptor-expressing immune cells to suppress inflammation [74]. Beyond its direct anti-inflammatory effects, α7 nAChRs activation has been implicated in tissue protection and repair mechanisms. Studies have shown that α7 nAChR activation can promote cell survival, angiogenesis, and tissue regeneration in various organs, including the lung [76], and brain [77], following injury or inflammation.

### Receptors of the KP and their roles in the pathophysiology of migraine

The aforementioned receptors play pivotal roles in migraine pathomechanisms, as summarized previously [78]. Glutamate and its receptors are integral to the pathophysiological processes underlying migraine. Elevated extracellular glutamate levels can precipitate cortical spreading depression [79], a phenomenon considered the neurophysiological basis for migraine aura and implicated in activating migraine pain pathways. Additionally, these receptors are localized in the trigeminal ganglion (TRG) and central terminals of trigeminal neurons [80]. Activation of glutamate receptors heightens neuronal excitability and neurotransmitter release, thereby facilitating transmission and amplification of pain signals.

Concurrently, AhR receptors are emerging as significant contributors in the intricate landscape of migraine pathophysiology. They influence chronic migraine development by modulating T cells in rodent models [81]. Furthermore, GPR35 activation has been shown to regulate the secretion of pro-inflammatory cytokines [54], potentially modulating the inflammatory environment linked to migraine. Additionally, GPR35 modulates pain perception [82], influencing nociceptor function and pain signaling [82, 83], presenting it as a potential target for pain management in migraine patients. Moreover, α7 nAChR, known for its anti-inflammatory properties, may alleviate inflammatory responses associated with migraine. Activation of α7 nAChR has the potential to diminish the release of inflammatory mediators from glial cells and neurons [84], contributing to the mitigation of migraine symptoms. Furthermore, α7 nAChR modulates pain pathways and is expressed in brain regions implicated in pain processing, including the TG [85], pivotal in migraine pathophysiology.

### The involvement of the KP in inflammation as a potential pathomechanism in migraine

Inflammation indeed plays a significant role in the pathomechanism of migraine, contributing to the initiation, propagation, and persistence of migraine attacks. Activation of trigeminal nerve and its branches releases neuropeptides such as substance P, CGRP, and neurokinin A [86, 87]. These neuropeptides cause vasodilation and inflammation of blood vessels, leading to pain and other migraine symptoms. On the other hand, during migraine attacks, there is an increase in the release of pro-inflammatory cytokines such as IL-1β, IL-6, and TNF-α [88]. These cytokines can sensitize pain receptors and promote neuroinflammation, exacerbating migraine symptoms. In addition to this, inflammation can disrupt the blood-brain barrier (BBB), which normally regulates the passage of substances between the bloodstream and the brain. BBB dysfunction allows inflammatory mediators and immune cells to enter the brain, further promoting neuroinflammation and contributing to migraine pathophysiology [89].

While its exact role of inflammation in migraine is still being elucidated, emerging research suggests that the KP and its downstream metabolites may contribute to migraine pathophysiology through various mechanisms. Kynurenine metabolites, particularly KYNA and QUIN, have been implicated in neuroinflammatory processes [90]. These metabolites can modulate the activity of immune cells, such as microglia and astrocytes [91], leading to the release of pro-inflammatory cytokines and chemokines in the CNS.

Multiple animal models exist for studying migraine [92]. Previous studies conducted by our research group demonstrated that the nitric oxide donor nitroglycerin (NTG) can elevate the protein levels of NF-κB, and cyclooxygenase-2 (COX-2) in rats, which are recognized as inflammatory markers [93]. Furthermore, NTG has been observed to influence numerous crucial enzymes within the KP in this particular model [94]. Another trigeminal activation model involves applying inflammatory agents to the dura mater, replicating neurogenic inflammation observed in rodents, which is pivotal in the pathomechanism of migraine. Commonly utilized for this purpose are Complete Freund’s Adjuvant (CFA) and a blend of inflammatory mediators known as inflammatory soup (IS). Previously, we demonstrated that applying IS to the dura mater induces a more intense, short-term c-Fos activation compared to CFA [95]. Additionally, our research revealed that both sumatriptan and KYNA can mitigate the effects induced by IS [96], confirming the involvement of the KP in neurogenic inflammation within a trigeminal activation model. CFA is additionally employed to chemically stimulate the orofacial area in animals probably by inducing inflammation [97]. In this model, we found altered KP components, providing further evidence that the KP has a role in trigeminal activation and inflammation [98]. On the other hand, inducing dural inflammation with an IS and CFA has been demonstrated to provoke migraine-like symptoms, including periorbital sensitivity [99, 100]. Another model used to study trigeminal pain is the orofacial formalin or CFA model that can induce a biphasic nociceptive response and inflammation [101]. In this model, our research group has demonstrated the effectiveness of KYNA analogue and probenecid in reducing both the immunohistochemical and behavioral alterations induced by formalin [102, 103]. In summarizing the animal data, various trigeminal activation models have effectively employed the KP substances to mitigate inflammatory processes.

While animal studies show promising results, it is important to emphasize that these findings may not directly translate to humans. Although the animal data suggest the potential for future drug therapies, developing these therapies will require extensive human research.

The KP is closely linked to the production of ROS and oxidative stress. Elevated levels of kynurenine metabolites have been associated with increased oxidative damage in various neurological disorders [104], including migraine [105]. Oxidative stress can exacerbate neuroinflammation, promote neuronal dysfunction, and contribute to migraine pathophysiology. The KP has immunomodulatory effects, influencing the function of immune cells and the production of inflammatory mediators through their receptors. Immune-mediated processes may contribute to neuroinflammation and migraine susceptibility. Altered Trp metabolism and the KP activity have been observed in patients with different types of headaches [106–109], suggesting a potential link between immune dysregulation and migraine pathogenesis. Targeting the KP has emerged as a potential therapeutic strategy for migraine treatment. Modulating the levels or activity of kynurenine metabolites, such as by using enzyme inhibitors or receptor antagonists, might help alleviate neuroinflammation and reduce migraine severity. In summary, the KP and its metabolites play complex roles in the inflammation associated with migraine. Further research into the specific mechanisms underlying these effects could lead to the development of novel therapeutic approaches for migraine management.

Understanding the role of inflammation in migraine pathophysiology has led to the development of novel treatment approaches targeting inflammatory pathways. For instance, medications that block CGRP or its receptors have shown efficacy in migraine prevention by reducing neurogenic inflammation. Additionally, lifestyle modifications aimed at reducing inflammation, such as stress management and dietary changes, may complement pharmacological interventions in migraine management.

### Inhibiting neuropeptides: a new frontline in migraine therapy

In recent years, research on migraine has focused on neuropeptides as potential contributors to the condition’s causes and as promising therapeutic options. Newly approved CGRP mAbs exhibit responder rates of 27–71%, while CGRP receptor inhibitors show rates ranging from 56 to 71%. To address the need for new therapeutic avenues, researchers are investigating the potential of PACAP from the secretin family, as a groundbreaking migraine treatment. Preclinical models have revealed PACAP’s impact on the trigeminal system, which is implicated in headache disorders. A previous study indicates that electrical stimulation of trigeminal ganglion (ES-TRG) led to a significant increase in PACAP1–38 immunoreactivity in the plasma 180 min post-treatment, and elevated levels of PACAP1–38 and PACAP1–27 immunoreactivity in the nucleus trigeminus caudalis (TNC). Apart from ES-TRG, intraperitoneal administration of NTG also triggered a rise in PACAP1–38 and PACAP1–27 expression in the TNC 180 min after treatment [12]. Another study demonstrated that administering PACAP1–38 resulted in heightened CGRP expression in the TNC, suggesting a possible correlation between CGRP release and PACAP1–38 [110]. These results are consistent with our previous study; following TS activation, there is a simultaneous increase in the release of PACAP and CGRP. Overexpression of neuropeptides correlated with mechanical allodynia [97].

Repeated electrical stimulation of the dura mater led to increased expression levels of CGRP and PACAP in the TRG and TNC of rats, with variations depending on the duration of stimulation (1, 3, and 7 days). This indicates that the frequency of stimulations may impact the release and actions of neuropeptides [111]. Activated microglial cells were observed in the ipsilateral TNC and cervical dorsal horn 72 h after administering orofacial CFA treatment to rats. These activated microglial cells might play a role in central sensitization and nociception mechanisms [112–114]. There is evidence suggesting that an antagonist of the P2 × 4 microglial receptor inhibited NTG-induced c-Fos expression and CGRP release in the TNC, subsequently alleviating hyperalgesia [115].

The endogenous antagonists of the NMDA receptor, KYNA, and its synthetic analog SZR-72, demonstrated the ability to inhibit the overexpression of PACAP at both the proteome and transcriptome levels. Another interesting finding of this study is that the expression levels of PACAP are significantly different between the noncompetitive NMDA receptor antagonist MK-801-, and the SZR-72-treated groups, which raises the possibility of the involvement of additional KYNA targets besides NMDA. Apart from NMDA glutamate receptor, studies indicate that KYNA also influences AMPA, kainate, AchRs, G protein-coupled, and opiate receptors. This suggests that KYNA and SZR-72 hold promise as potential new drug candidates for PACAP-targeted migraine therapy in the future [19, 116].

### Investigating potential drawbacks of targeting the KP in migraine treatment

The KP has garnered attention in migraine research due to its potential roles in neuroinflammation and pain modulation, as we discussed earlier. While much of the focus has been on its therapeutic potential, there are considerations regarding negative data and potential side effects associated with targeting this pathway in migraine treatment. Some metabolites within the KP, such as QUIN, are neurotoxic at high concentrations and can contribute to neuronal damage and neuroinflammation [117, 118]. It follows that elevated levels of neurotoxic metabolites could potentially exacerbate neurological symptoms in migraine patients. Pharmacological targeting of specific enzymes or receptors within the KP may lead to unintended side effects. For example, drugs designed to modulate KP activity could affect other physiological processes, impacting overall health and potentially exacerbating migraine symptoms. On the other hand, responses to the KP modulation can vary among individuals. Genetic factors, environmental influences, and variations in the KP metabolite levels may contribute to differing responses and potential side effects in migraine patients. Despite promising preclinical and clinical findings [117], clinical trials targeting the KP in migraine are limited. The efficacy and safety of the KP-targeted therapies specifically for migraine management are still under investigation, and more research is needed to fully understand their potential negative effects. However, it should be noted that there are several KYNA analogues known not to convert to QUIN. Therefore, further studies with these analogues may be the way forward [117].

## Conclusion

**Fig. 4:**
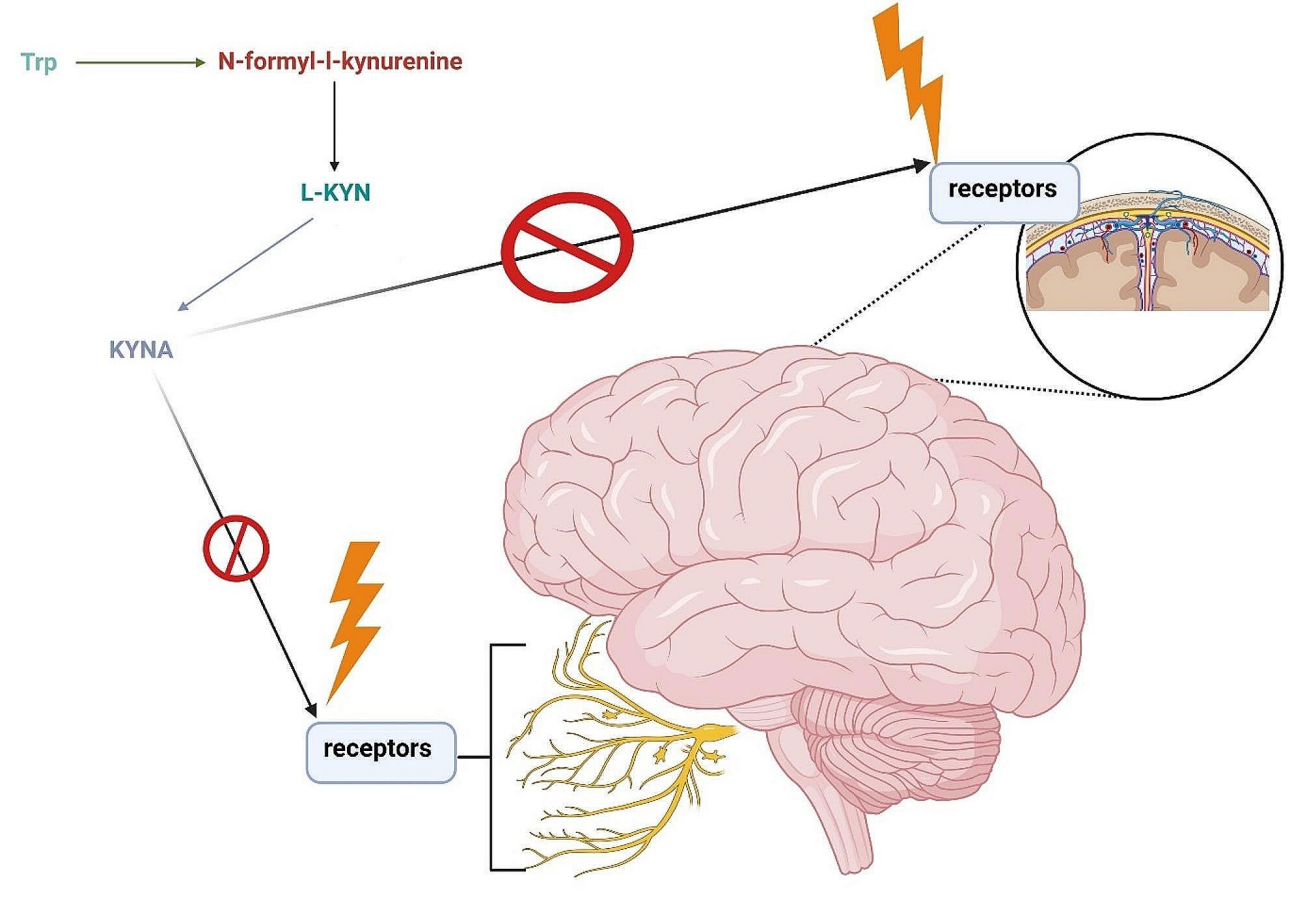
This figure outlines the potential targets of the KP within the nervous system. KP receptors are distributed across both the central and peripheral nervous systems, indicating that potential targets of the KP are found in both regions. Abbreviations: KYNA—kynurenic acid, L-KYN—L-kynurenine, Trp—tryptophan

In summary, the KP plays a crucial role in the pathomechanism of migraine (Fig. 4) [Insert Fig. 4]. KP receptors are not only present in the nervous system but also in the immune system, suggesting potential effects of KP in both domains [118]. Although the exact role of the immune system in migraine development remains incompletely understood, inflammation is undoubtedly implicated in migraine pathogenesis. Currently, migraine treatment revolves around neuropeptides, which are signaling molecules produced by neurons, can modulate various aspects of immune function. They can act as immunomodulators, influencing the activity of immune cells and can regulate the release of cytokines. On the other hand, the immune system can also influence neuropeptide activity by regulating their synthesis and release. This bidirectional communication between neuropeptides and the immune system plays a crucial role in various physiological processes, including inflammation, and pain modulation. Dysregulation of this communication has been implicated in the pathogenesis of numerous diseases, including autoimmune disorders, inflammatory conditions, and neurological disorders such as migraine.

## Data Availability

No datasets were generated or analysed during the current study.

## Abbreviations

3-HA: 3-hydroxyanthranilic acid
3-HAO: Kynureninase
3-HK: 3-hydroxykynurenine
AhR: Aryl hydrocarbon receptor
ANA: Anthranilic acid
BBB: Blood brain barrier
CFA: Complete Freund’s Adjuvant
CGRP: Calcitonin gene-related peptide
CNS: Central nervous system
COX-2: Cyclooxygenase-2
GPR35: G protein-coupled receptor 35
IDO: Indoleamine 2,3-dioxygenase
IFN-γ: Interferon-gamma
IS: Inflammatory soup
KATs: Kynurenine aminotransferases
KMO: Kynurenine 3-monooxygenase
KP: Kynurenine pathway
KYNA: Kynurenic acid
LC: Locus coeruleus
L-KYN: L-kynurenine
mAb: Monoclonal antibody
NAD+: Nicotinamide adenine dinucleotide
NF-κB: Nuclear factor-kappa B
NMDA: N-methyl D-aspartate
NTG: Nitroglycerin
PAC1: PACAP receptor 1
PACAP: Pituitary adenylate cyclase activating polypeptide
PAG: Periaqueductal grey
QUIN: Quinolinic acid
ROS: Reactive oxygen species
SSC: Somatosensory cortex
TH: Thalamus
TNC: Nucleus trigeminus caudalis
TRG: Trigeminal ganglion
Trp: Tryptohpan
TS: Trigeminovascular system
α7 nACh: α-7 nicotinic acetylcholine
